# Sub lethal levels of platinum nanoparticle cures plasmid and in combination with carbapenem, curtails carbapenem resistant *Escherichia coli*

**DOI:** 10.1038/s41598-019-41489-3

**Published:** 2019-03-28

**Authors:** Subhashree Bharathan, Niranjana Sri Sundaramoorthy, Harini Chandrasekaran, Gagana Rangappa, GaneshPrasad ArunKumar, Siva Bala Subramaniyan, Anbazhagan Veerappan, Saisubramanian Nagarajan

**Affiliations:** 1 Center for Research in Infectious Diseases, School of Chemical and Biotechnology, SASTRA Deemed to be University, Thanjavur, India; 2 Department of Chemistry, School of Chemical and Biotechnology, SASTRA Deemed to be University, Thanjavur, India

## Abstract

Drug resistance traits are rapidly disseminated across bacteria by horizontal gene transfer, especially through plasmids. Plasmid curing agents that are active both *in vitro* and *in vivo* will resensitize Multi Drug Resistant (MDR) bacteria to antimicrobial agents. Pectin capped platinum nanoparticles (PtNPs) at sub MIC (20 µM) concentration was effective, in causing loss of Extended Spectrum Beta Lactamase (ESBL) harboring plasmid as evidenced by, absence of plasmid in agarose gel and by a concomitant (16–64 fold) drop in MIC for cell wall inhibitors ceftriaxone and meropenem, in carbapenem resistant *Escherichia coli* (CREC). Interestingly, the plasmid cured strain exhibited small colony morphology and displayed slower growth both *in vitro* and *in vivo*. Complementation of cured strain with plasmid from the wild type strain restored resistance towards meropenem and ceftriaxone. Relative to wild type, plasmid cured strain displayed 50% reduction in biofilm formation. Plasmid curing also occurred *in vivo* in infected zebrafish with curing efficiency of 17% for nanoparticle + meropenem treatment. PtNPs + meropenem reduced bioburden of CREC in infected zebrafish by 2.4 log CFU. Mechanistic studies revealed that nanoparticle interacted with cell surface and perturbed inner membrane integrity. PtNPs did not induce ROS, yet it caused plasmid DNA cleavage, as evidenced by gyrase inhibition assay. Our study for the first time reveals that PtNPs as plasmid curing agent can resensitize MDR bacteria to selective antimicrobial agents *in vivo*.

## Introduction

Multi Drug Resistant (MDR) pathogens pose severe threat to public health, which has prompted WHO to declare 12 infectious agents as priority pathogens, urging researchers across the globe to devise urgent measures in the form of new antimicrobial agents or resistance modulatory agents to curtail these pathogens. Extended Spectrum Beta Lactamase (ESBL) harboring *Enterobacteriaceae* falls under the critical priority category. Presently, only a few drugs like colistin, tigecycline, fosfomycin, aminoglycosides etc are considered as last resort drugs for treating nosocomial infections caused by carbapenem resistant *Enterobacteriaceae*^1^. The situation gets exacerbated by the fact that lately, plasmid mediated resistance to colistin (last resort drug) is being widely reported in *E. coli* isolates from humans, animals and even the environment^2–5^, which implies wide spread dissemination of colistin resistant gene (mcr-1) by horizontal gene transfer (HGT)^6^. These reports underscore the importance of plasmid curing in curtailing spread of drug resistant genes across populations.

Even though drug resistant trait could be chromosomally harbored, in many instances resistance to third generation betalactams like cephalosporins, carbapenems in gram negative *Enterobacteriaceae* are typically mediated by plasmids such as *bla*_KPC_, *bla*_NDM-1_, *bla*_VIM_, *bla*_CTX-M_^7,8^. Hence apart from discovering novel antimicrobials agents to mitigate priority pathogens, plasmid curing agents could also be viewed as potential resistance modulatory agents.

Plasmid curing implies loss of plasmid from host strain due to treatment with various compounds. Plasmid elimination from the host occurs predominantly by two mechanisms (i) inhibition of plasmid replication (ii) interfering with plasmid segregation. On the other hand, inhibiting transconjugal transfer will avert the dissemination of plasmid by HGT across populations, but it will have least impact on vertical gene transfer^9^. Although many plasmid curing agents are very well known for a long time including intercalating agents like acridine orange^10^, ethidium bromide^11^ surfactants like SDS^10^, glycine^12^, heterocyclic organic compounds^9,13^ and certain plant metabolites like plumbagin^14^, most of these agents were only effective *in vitro*, either they were toxic *in vivo* or their efficacy as plasmid curing agents *in vivo* has not been explored earlier.

Metals in nanosize have been widely investigated to mitigate multi-drug resistant bacteria. We have reported earlier that casein capped copper nanoparticles (casCuNPs) combats methicillin resistance *Staphylococcus aureus*. Interestingly, at low concentration casCuNPs serves as an efflux pump inhibitor and prevents biofilm formation^15^. Although CuNPs are beneficial in terms of antimicrobial activity, it is highly toxic when tested in zebrafish animal model. Even the highly appreciated antimicrobial silver nanoparticles display toxicity in zebrafish. As a continuous effort, our group identified pectin capped platinum nanoparticles (PtNPs) for treating bacteria infected zebrafish and rescued them from infection without inducing any additional toxicity to the fish^16^. PtNPs not only rescues the fish from infection but also promotes adaptive immune response against the pathogen, so much so that zebrafish is able to survive repetitive infection^17^. Inspired from these studies, the plasmid curing effect of PtNPs was investigated for the first time. Hence the objective of the present study is to evaluate potential of sub MIC levels of PtNPs to cure plasmid *in vitro* and *in vivo* to curtail drug resistance and explore the mechanism of curing mediated by PtNPs. Our results showed that pectin capped platinum nanoparticle can function at sub Minimum Inhibitory Concentration (MIC) as plasmid curing agent in MDR clinical isolate of *E. coli* both *in vitro* and *in vivo* in a zebrafish infection model. PCR amplification revealed that plasmid harbored *bla*_NDM-5_, *bla*_OXA- 23_ and *bla*_OXA- 48_ ESBL genes, which could account for carbapenem resistance exhibited by the plasmid cured strain. Attempts to explore the mechanism of curing using various studies like TEM imaging, membrane permeability, Reactive Oxygen Species (ROS), membrane potential and membrane integrity indicate that sub MIC levels of PtNPs interacted with cell surface and compromised the inner membrane integrity without affecting cell viability. Gyrase inhibition assay revealed that PtNP treatment, both in presence and absence of gyrase, induced DNA cleavage even at sub MIC concentrations which might account for plasmid curing ability of PtNPs.

## Results and Discussion

### Pectin capped PtNPs against *E. coli*

Mucoadhesive property of pectin motivated us to use them in the synthesis of nanoparticles^18^. The aqueous mixture of hexachloroplatinic acid and pectin on reduction with sodium borohydride produces brownish black platinum nanoparticles (PtNPs)^19^. These NPs were stable for months without fading and aggregating in solution. The zeta potential of prepared PtNPs was −38.9 ±6.7 mV, indicating the electrical boundaries of the NPs are well separated and the capping agent pectin prevents them from aggregation. The size and morphology determined by transmission electron microscopy revealed that the particles are spherical with size range from 2–6 nm (Supplementary Fig. 1), which is consistent with the previous report.

After testing for their stability at ambient temperature for 7 days, PtNPs were evaluated for its antibacterial effect against the clinical isolate of *E. coli*. Based on drug resistance profile (Supplementary Table 1), U3790 strain was deemed as Extremely Drug Resistant (XDR) isolate as proposed earlier for classification of drug resistant strains^20^. Antibacterial effect of PtNPs against *E. coli* reference strain and U3790 strain was evaluated by microbroth two fold dilution method, which revealed that PtNPs exhibited a high MIC of >256 µM against both strains of *E. coli*.

### Plasmid curing effect of PtNPs – an *in vitro* study

**Figure 1:**
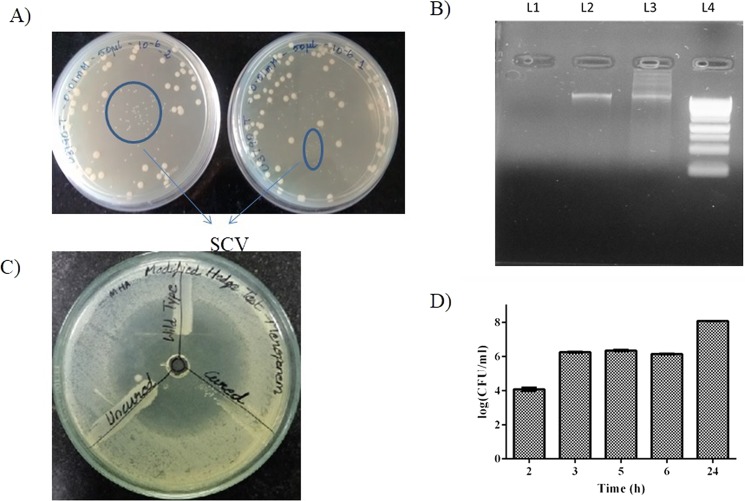
Treatment with pectin capped platinum NPs cures plasmid and causes formation of small colony variants (SCV) in plasmid cured *E.coli* strains, SCV exhibits meropenem sensitive phenotype. (**A**) Appearance of small colony variants (SCV) due to PtNP treatment. (**B**) Gel picture showing absence of plasmid in cured SCV strain (L1) and its presence in uncured strain (L2) and in wild type (L3), 1 KB ladder Molecular weight marker (L4). (The full length gel image is shown as Supplementary Fig. 2) (**C**) Cured SCV fails to grow near meropenem implying loss of plasmid resensitizes SCV to meropenem, (**D**) SCV starts appearing from 3 h post treatment with PtNPs. The experiment was performed in triplicates and the error bar indicates the standard error of the mean.

Preliminary analysis revealed that the MDR *E. coli* strain harbored a single plasmid >10 kb. Following treatment with PtNPs for 24 h, the culture was plated onto Luria Bertani (LB) agar and spot plated onto LB + meropenem. The colonies that grew on LB+ meropenem and the ones that failed to grow on meropenem containing plates were randomly picked and evaluated for the presence or absence of plasmid by plasmid DNA extraction followed by agarose gel electrophoresis. Our observations showed that consistently PtNPs treatment altered morphology of strains and strains that had lost the plasmid formed small colony variants (SCV) (Fig. 1A), whereas plasmid containing strains exhibited normal/ relatively large colony variant (LCV) phenotype (Fig. 1A). Repeated testing (at least 10 times with ~8 colonies during each testing) showed that almost all small colony variants (SCV) failed to exhibit growth upon respotting onto meropenem containing plates, indicating plasmid loss, which was further confirmed by absence of plasmid band on agarose gel (Fig. 1B and Supplementary Fig. 2). Modified Hodge test showed that cured strain (SCV) fails to grow near 10 µg meropenem disc, implying that it is sensitive to meropenem, whereas uncured strain harboring plasmid grows near meropenem disc showing that it is resistant to meropenem (Fig. 1C). Antibiotic sensitivity test (ABST) test showed that plasmid cured SCV strain displayed a larger zone of inhibition (16 mm) compared to wild type strain which displayed a relatively smaller zone of inhibition (10 mm) (Supplementary Fig. 3). Most importantly, plasmid loss was accompanied by a 16 fold reduction in ceftriaxone MIC (128 µg to 8 µg/ml) and a remarkable 64 fold reduction in meropenem MIC (32 µg to 0.5 µg/ml), which is well below the CLSI break point >4 µg/ml proving that plasmid loss leads to resensitization of carbapenem resistant *E. coli* to meropenem *in vitro*. Among the other antimicrobials tested, plasmid loss correlated with a 4 fold drop in MIC for streptomycin, tobramycin and tetracycline (Table 1), although the reduced concentrations for these protein synthesis inhibitors were still above CLSI breakpoints for the respective antimicrobial agents. We were curious to identify ESBL genes that were harbored in the plasmid, towards this end we extracted plasmid and performed a PCR amplification of ESBL genes using plasmid DNA as template and our results showed that plasmid harbored *bla*_OXA-23_, *bla*_OXA-48_ and *bla*_NDM_ genes (Supplementary Fig. 4). Partial plasmid sequences were obtained by sequencing using MySeq platform. The sequences were assembled into contigs and subjected to analysis using plasmid finder tool of center for genomic epidemiology. Based on plasmid types that were identified by plasmid finder tool, specific primers were designed  and PCR amplification was carried out using plasmid DNA as template. The results revealed that plasmid belonged to following incompatability groups IncI1, IncF1a, IncF1b, IncFII and pO111 (Supplementary Table 2 and Supplementary Fig. 5). Attempts to employ SDS/ acridine orange as control for plasmid curing experiments were unsuccessful (data not shown). Therefore we reasoned that U3790 might possess a capsule, which might have hindered ability of SDS/acridine orange to eliminate plasmid. Capsular staining revealed that as expected, U3790 does have a capsule (Supplementary Fig. 6). Presence of capsular polysaccharide has been widely reported among clinical isolates of *E. coli* and it was shown to confer survival advantage when grown on whole blood^21,22^.

**Table 1:** Treatment with PtNPs cures plasmid and is accompanied by a drop in MIC for select cell wall inhibitors and protein synthesis inhibitors.

Antibiotics	Minimum Inhibitory Concentration (µg/ml)		Modulation factor
	Wild	Cured SCV*	
Streptomycin	64	16	4
Tobramycin	128	32	4
Gentamycin	>128	>128	1
Ciprofloxacin	>128	>128	1
Tetracycline	128	32	4
Meropenem	>32	0.5	>64
Ceftriaxone	>128	8	>16

*SCV – Small colony variant that has lost its plasmid.

Among treatment ranges evaluated (viz., 1, 5, 10, 20 and 30 μM), plasmid elimination was observed from 10 μM concentration (Table 2) and at 20 μM concentration, PtNPs caused effective loss of plasmid, as evidenced by absence of plasmid band from the cured strain relative to both wild type and uncured strain on 1% agarose gel (Fig. 1B and Supplementary Fig. 2). In order to discern time required for PtNPs to affect plasmid curing, bacteria were treated with 20 μM of PtNPs and at different time points (2, 3, 5, 6 & 24 h), cells were plated onto LB to discern total count and on LB+ meropenem to glean count of plasmid retaining colonies. The frequency of appearance of SCV (plasmid cured strain), as confirmed by its inability to grow on LB+ meropenem was quantified. The SCVs were also randomly checked for absence of plasmid by agarose gel (data not shown). The results (Fig. 1D) revealed that plasmid curing due to PtNP treatment occurs from 3 h with a curing efficiency of 17.19% (Table 3). At subsequent time points, curing efficiency decreased to 14.86% by 5 h and it further dropped to 10.51% by 24 h, which could be due to remodeling of bacterial cell wall in stationary phase^23^ or sequestration of PtNPs by cells/ cell debris in stationary phase. Importantly, treatment with Triton-X-100 over the time course did not result in plasmid curing and frequency of appearance of plasmid lost, small colony variant in Triton-X-100 treatment was comparable to that arising spontaneously in untreated control with curing efficiency ranging at ~1% by 24 h (Table 3).

**Table 2:** Concentration dependent curing showed that plasmid curing occurs from 10 µM.

Concentration of PtNPs	No. of Small Colonies	No. of Large Colonies	Total No. of Colonies	% Large Colonies	% Small colonies
0.001 mM	2 ± 1	40 ± 11	41 ± 13	97 ± 4.24	3 ± 4.24
0.005 mM	1 ± 1	201 ± 83	202 ± 83	99 ± 0.49	1 ± 0.49
0.01 mM	31 ± 4	51 ± 25	82 ± 21	60 ± 15.25	40 ± 15.25
0.02 mM	76 ± 9	106 ± 25	182 ± 16	58.00 ± 8.83	41.99 ± 8.83

**Table 3:** Plasmid curing occurs from 3 h with PtNP and not with Triton X 100 treatment.

Time (h)	Untreated			PtNPs treated			Triton X-100 treated		
	No. of cured colonies	Total no. of colonies	Relative % of cured colonies	No. of cured colonies	Total no. of colonies	Relative % of cured colonies	No. of cured colonies	Total no. of colonies	Relative % of cured colonies
**2**	3 ± 2	322 ± 25	**0.90** ± **0.55**	6 ± 1	824 ± 51	**0.72** ± **0.08**	8 ± 6	641 ± 100	1.17 ± 0.76
**3**	4 ± 1	285 ± 10	**1.4** ± **0.3**	79 ± 19	454 ± 57	**17.19** ± **1.98**	3 ± 2	433 ± 35	0.67 ± 0.41
**5**	6 ± 1	987 ± 26	**0.61** ± **0.09**	123 ± 21	824 ± 46	**14.86** ± **1.72**	20 ± 5	864 ± 74	2.29 ± 0.38
**24**	7 ± 1	479 ± 33	**1.46** ± **0.11**	65 ± 9	617 ± 65	**10.51** ± **0.35**	3 ± 1	402 ± 65	0.73 ± 0.13

**Figure 2:**
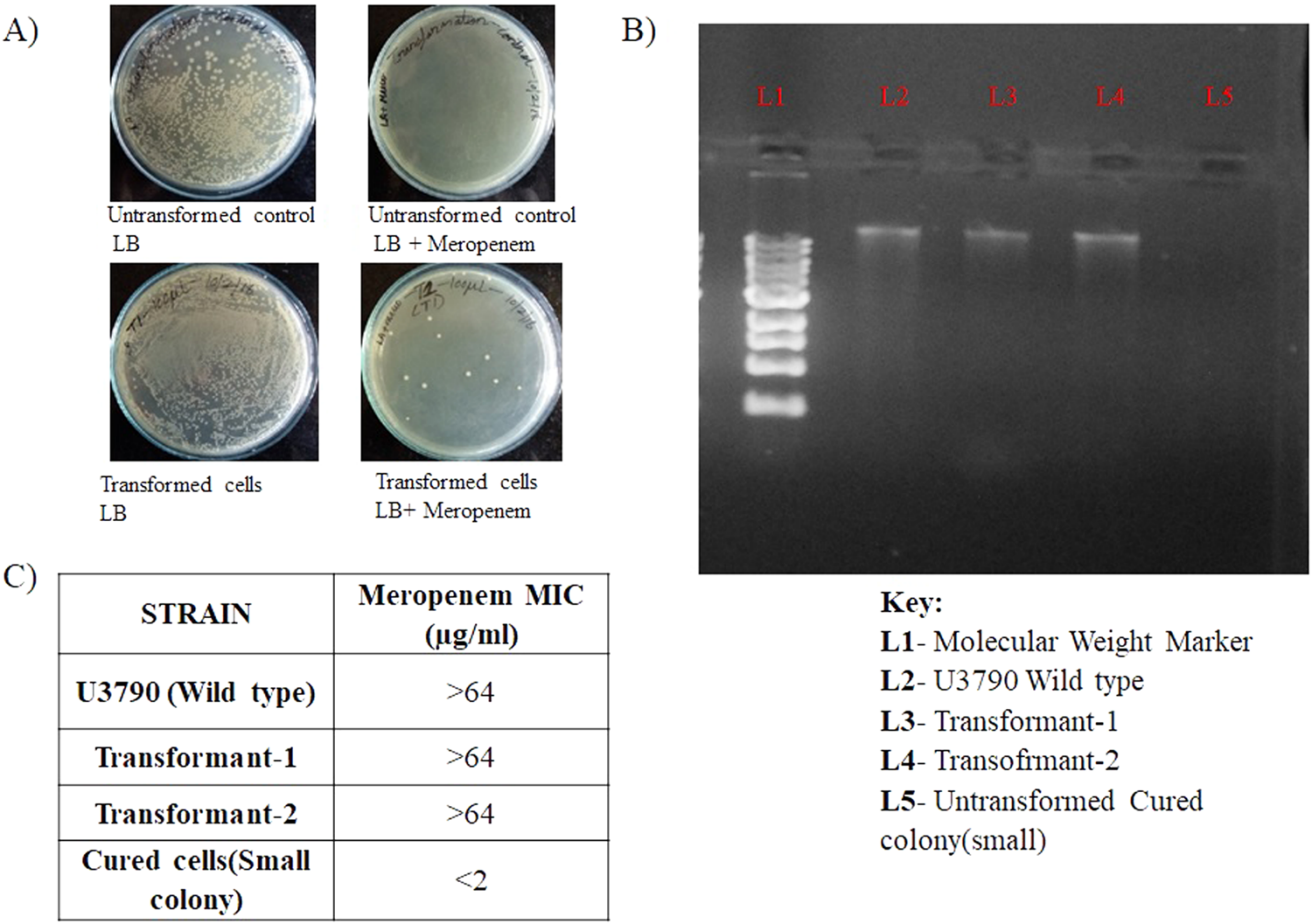
SCV transformed with plasmid exhibits elevated MIC for meropenem. (**A**) Only the transformants grew on LB with meropenem. (**B**) Plasmid extracted from transformants on LB+ antibiotic plate showed reappearance of plasmid band (The full length gel image is shown as Supplementary Fig. 7) and (**C**) the transformed strain exhibited elevated MIC for meropenem.

To confirm whether the drug sensitive phenotype of the small colony variant (cured strain) was due to the plasmid, we complemented the cured SCV strain with plasmid from wild type U3790 strain, by the conventional calcium chloride mediated transformation. The transformed strains were selected on plates containing meropenem, which permitted growth of plasmid harboring strain and inhibited growth of the strain that lacked the plasmid. The transformants showed the reappearance of plasmid band on agarose gel and regained resistance to meropenem (Fig. 2 and Supplementary Fig. 7) proving that meropenem resistant phenotype was indeed conferred by the plasmid.

Having established optimal concentration for curing, we tested whether PtNPs mediated curing can also occur in another laboratory strain of *E. coli*, DH5α strain harboring yeast expression plasmid p2GB42/Kan with a kanamycin resistant marker. We also employed SDS and acridine orange as controls, Results showed that treatment with either PtNPs or positive controls SDS/acridine orange, successfully eliminated plasmid from the *E.coli* strain as evidenced by absence of band corresponding to plasmid on agarose gel and inability of plasmid cured colonies to grow on kanamycin containing plates when respotted (Supplementary Fig. 8).

Small colony phenotype of cured *E. coli* strain indicated that the plasmid loss was accompanied by slower growth relative to wild type or uncured strain. In other words, plasmid might harbor some nutrient acquisition genes. Because plasmid loss conferred a slow growing phenotype on LB agar plates, we performed the growth fitness assay of SCV relative to wild type strain that harbored the plasmid in dilute LB. The results showed that until day 6, both SCV and LCV exhibited comparable cell counts, by day 7, a steep decline in SCV population was observed (Supplementary Fig. 9) indicating that SCV exhibited relatively lower growth fitness relative to wild type strain harboring plasmid only by day 7, and at earlier time points fitness disadvantage was not seen between SCV and LCV.

**Figure 3:**
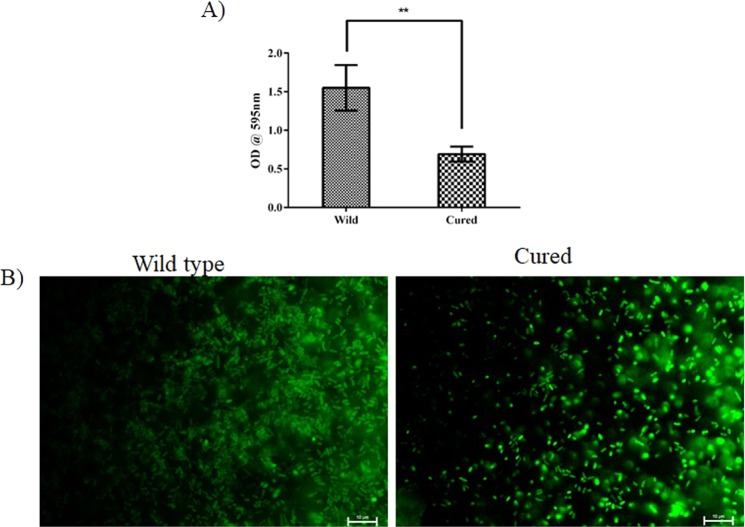
Plasmid curing causes decline in biofilm forming ability in cured U3790 strain relative to wild type plasmid harboring strain. (**A**) Biofilm formation in wild type and cured strain quantified by crystal violet assay was found to be statistically significant with P = 0027. (**B**) The biofilms formed by wild type and plasmid cured strain were stained with Syto9 stain and imaged using fluorescent microscope at 100X magnification.

We evaluated if plasmid cured strain differed from wild type strain in its biofilm forming ability. Crystal violet assay revealed that quite unexpectedly, plasmid cured strain displayed 50% reduction in biofilm forming ability relative to strain containing wild type plasmid (Fig. 3). In order to qualitatively evaluate relative biofilm forming capacity of wild type and plasmid cured strains, 24 h biofilms were formed on glass slide and stained with Syto9 and imaged using fluorescent microscope (Nikon Eclipsce Ni-U, Nikon, Japan). Fluorescent imaging revealed a drastic reduction in biofilm forming ability in the plasmid cured strain (Fig. 3B), which shows that plasmid curing apart from reversing drug resistance also curbs biofilm formation, which is an added advantage.

Our *in vitro* studies showed that PtNPs can cure plasmid from capsulated clinical strain of *E. coli*. Curing results in formation of SCV accompanied by a drop in MIC for meropenem and ceftriaxone. Complementation of plasmid retrieves antibiotic resistant phenotype and plasmid curing curtails biofilm forming ability of plasmid lost strain.

### Biocompatibility of PtNPs

The biocompatibility of PtNPs was evaluated using red blood cells (RBC). Exposing the RBC to 200 μM of PtNPs did not affect the morphology of RBC, indeed it remained like an untreated one and the membranes remained intact (Supplementary Fig. 10). Previous studies have shown that even at 0.5 mM, PtNPs was non-toxic to experimental zebrafish^17,19^. These results supports the observation that the PtNPs even at 25 times higher concentration employed for plasmid curing is non-toxic^16^.

### Plasmid curing effect of PtNPs – an *in vivo* study

Zebrafish was chosen as an *in vivo* model because of its 80% genetic homology to humans, coupled with its ease of growth and maintenance. Earlier work from our group has shown zebrafish serves as a better model to study bacterial infections prior to evaluation in higher animal models^17,24,25^. To evaluate whether plasmid curing can occur *in vivo*, fish were injected intramuscularly with 0.1 OD (corresponding to ~1 × 10^6^ Colony forming units (CFU)/ml) of U3790 strain and were segregated into four groups. Group A was treated with PtNPs alone, Group B was treated with meropenem alone and Group C was treated with PtNPs in combination with meropenem. Group D was untreated control. 48 h post infection, fish was anaesthetized using ms-222 and euthanized by decapitation. Muscle tissue was dissected, minced in sterile saline solution and was serially diluted and plated onto LBA. Colonies, especially small colony variants from LBA were respotted onto LB with meropenem, wherein absence of growth indicates loss of plasmid, which was confirmed by absence of plasmid band in agarose gel. Our results revealed that PtNPs indeed caused loss of plasmid *in vivo* in the fish tissue as evidenced by inability of fraction of isolates to grow on meropenem containing plates. Similar to *in vitro* treatment, NP treatment *in vivo* resulted in the formation of small colony variants and plasmid band was absent in SCV isolated from fish tissue after treatment with PtNP (Supplementary Fig. 11). The curing efficiency of meropenem + PtNP *in vivo* ranged around 17% (Table 4).

**Table 4:** PtNPs cures plasmid *in vivo* from infected muscle tissue of zebrafish.

Treatment	SCV (Colony Counts)	LCV (Colony Counts)	Total no of colonies	Curing efficiency (%)
Untreated	0	95 ± 26	95 ± 26	0
Meropenem	0	111 ± 44	111 ± 44	0
PtNPs	3 ± 2	74 ± 6	80 ± 11	3.65 ± 2.1
Meropenem + PtNPs	29 ± 1	110 ± 35	139 ± 26	16.96 ± 5.05

**Figure 4:**
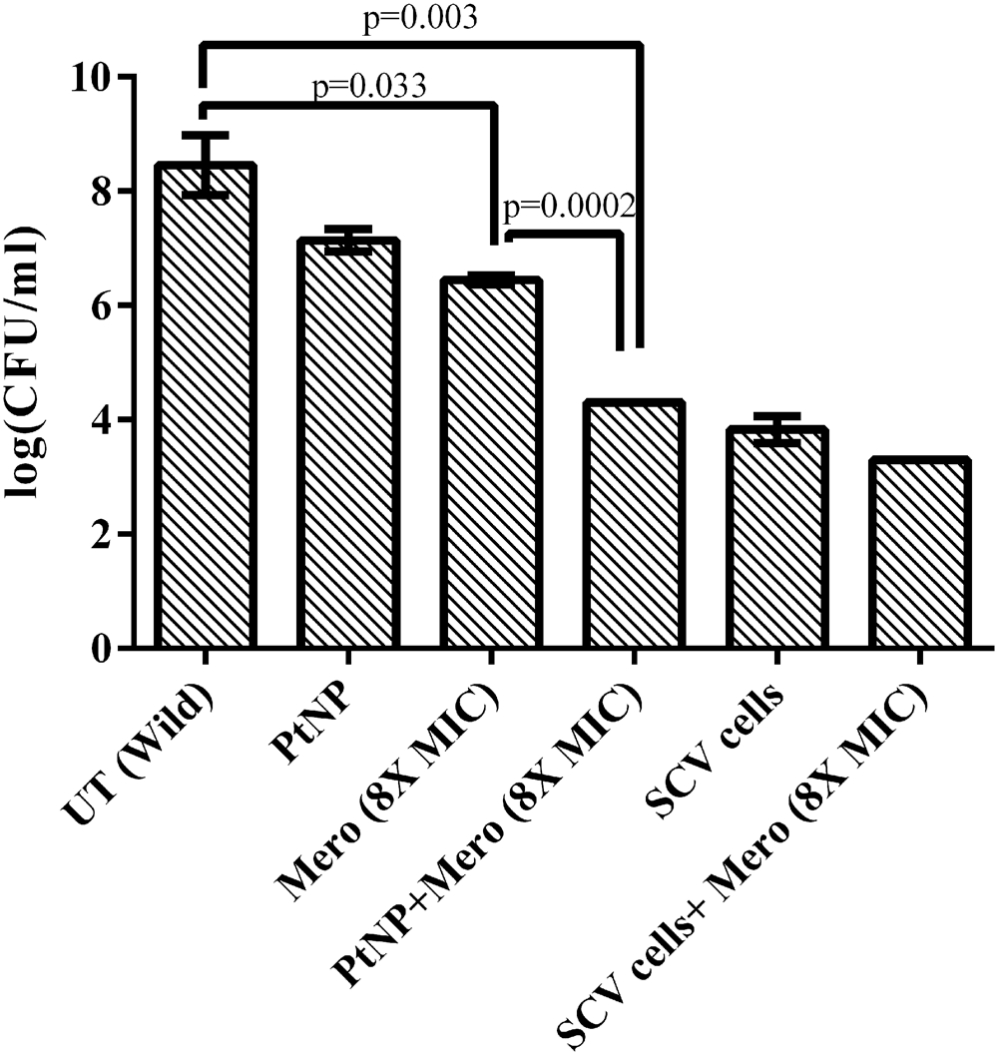
PtNP causes a remarkable reduction in bacterial bioburden of plasmid harboring strain in Zebrafish. Meropenem in combination with PtNPs caused ~2.4 log decline in bacterial CFU relative to meropenem treatment alone (P = 0.0002). Plasmid cured SCV fails to respond to Meropenem.

Having confirmed ability of PtNPs to cure *in vivo*, we explored the ability of PtNPs to act as an adjuvant to curtail growth of U3790 *E. coli* strain *in vivo*. Zebrafish was infected with either wild type or cured small colony variant. We observed that both strains could establish infection well. But small colony variant exhibited a lower cell density that is 4.5 log fold lower than that attained by wild type (Fig. 4), proving that plasmid confers growth advantage both *in vitro* and *in vivo* and its absence results in reduced growth. Wild type strain harboring plasmid reached a cell density corresponding to 10^8^ cells *in vivo* (Fig. 4). Treatment with 8X MIC of meropenem caused ~2 log decline for wild type strain, whereas SCV treated with 8X MIC of meropenem exhibited only a minor decline of 0.5 log CFU, which could be attributed to slow growth that confers resistance to antimicrobial agents. Treatment with sub MIC levels of PtNPs alone caused ~1 log decline in CFU of wild type relative to untreated control (Fig. 4). Importantly, treatment with a combination of PtNP and meropenem caused a drastic 5 log decline relative to untreated control and a significant 2.4 log decline relative to meropenem treatment alone, which shows that PtNPs by virtue of its plasmid curing efficiency, resensitizes carbapenem resistant *E. coli* strain to meropenem *in vivo* and thus NPs function as an adjuvant along with antibiotic in curtailing growth of MDR *E. coli in vivo* (Fig. 4). To the best of our knowledge ours is the first report to show that metal NPs at sub MIC levels can cure plasmid *in vivo*.

### Mechanism of action of PtNPs against drug resistance *E. coli*

**Figure 5:**
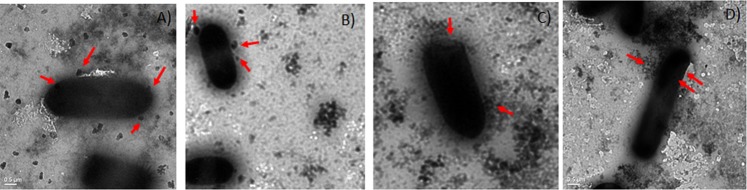
PtNPs interact with cell surface of *E.coli* either individually or as aggregates without damaging cell wall.

TEM imaging of interaction of bacteria with PtNPs was carried out without dehydration and fixation steps to prevent loss of capsule to discern if PtNPs were capable of penetrating the capsule. Our results revealed that PtNPs penetrates the capsule (which is observed as a hue around bacteria) and interacts with cell surface of bacteria, without causing damage to bacterial cell wall (Fig. 5). Further mechanistic explorations revealed that NPs did not cause a disruption of membrane potential, whereas positive control CCCP perturbed membrane potential in *E. coli* (Supplementary Fig. 12). Similarly PtNPs treatment did not affect outer membrane permeability in *E. coli* as revealed by NPN assay (data not shown). ROS assay also revealed that at concentration tested, PtNPs fails to induce ROS in *E. coli* (Supplementary Fig. 13). Interestingly, membrane integrity assay showed that PtNPs treatment caused perturbation of inner membrane with leakage of nucleic acid similar to 48% leakage seen with Triton-X-100 treatment and leakage of protein comparable to 29% of that observed in Triton- X- 100 treatment (Table 5). In order to check if perturbation in inner membrane integrity is accompanied by loss of viability, live dead staining and in parallel plating of treated cells were carried out. The results (Supplementary Fig. 14) revealed that NP treatment did not induce cell death and colony counts were comparable to untreated control. TEM imaging (Fig. 5) also supports the conclusion that PtNPs does not cause damage to cells and hence is unlikely to adversely affect the viability. But Triton- X- 100 treatment caused a significant proportion of dead cells and a marked decline in plate counts (Supplementary Fig. 14). Thus NPs treatment by 24 h causes leakage of nucleic acid without affecting cell viability.

**Table 5:** PtNP affects membrane integrity in *E.coli and* causes leakage of Nucleic acids.

Time (hr)	Untreated		PtNPs (0.02 mM)		Triton X-100 treatment (0.5% V/V)	
	Absorbance 260 nm	Absorbance 280 nm	Absorbance 260 nm	Absorbance 280 nm	Absorbance 260 nm	Absorbance 280 nm
1	0.193 ± 0.06	0.130 ± 0.04	0.622 ± 0.08	0.385 ± 0.05	3.047 ± 0.05	2.99 ± 0.11
2	0.248 ± 0.03	0.151 ± 0.04	0.543 ± 0.21	0.251 ± 0.17	3.053 ± 0.15	2.735 ± 0.21
3	0.274 ± 0.01	0.173 ± 0.02	0.690 ± 0.14	0.392 ± 0.07	3.113 ± 0.13	2.820 ± 0.08
4	0.293 ± 0.03	0.228 ± 0.07	0.722 ± 0.01	0.433 ± 0.03	3.137 ± 0.12	2.811 ± 0.11
24	0.48 ± 0.03	0.340 ± 0.02	1.590 ± 0.46	0.889 ± 0.12	3.309 ± 0.08	3.015 ± 0.03

**Figure 6:**
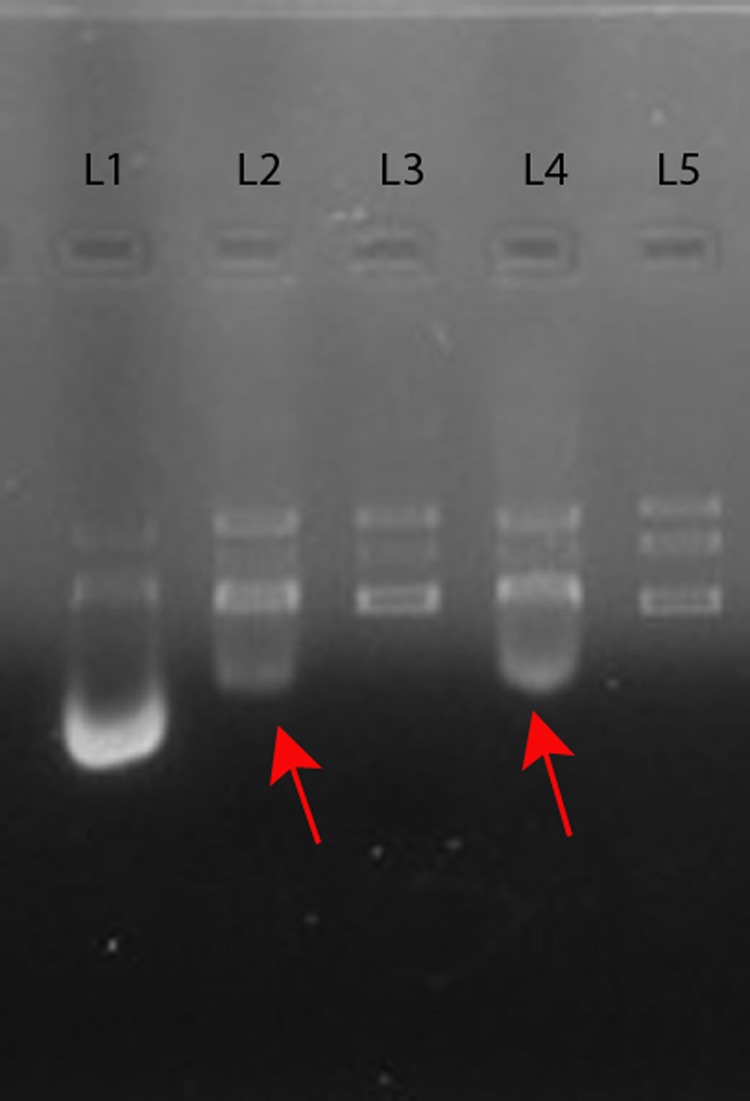
PtNPs induce DNA cleavage in presence and absence of gyrase. Different reactions were setup: Relaxed pHOT-1 DNA (substrate) with (i) gyrase and Ciprofloxacin (positive control) (ii) gyrase and PtNPs (iii) gyrase (iv) PtNPs. The samples were loaded on 1% agarose gel with appropriate markers electrophoresed and imaged and a representative gel image is presented here. The full length gel image is shown as Supplementary Fig. 15). Red arrows indicate cleaved DNA generated due to PtNP treatment. Lane Description: L1 – Relaxed DNA + gyrase + Ciprofloxacin; L2 – Relaxed DNA + gyrase + PtNPs; L3- Relaxed DNA + gyrase; L4 – Relaxed DNA + PtNPs; L5 – Relaxed DNA.

Most plasmid curing agents affects either replication or segregation of the plasmid. Planar molecules serve as good intercalating agent. An earlier report proposed that Cis dichlorodiamine platimum (II) chloride by virtue of its planar structure probably acts as an intercalating agent and cured ColE1 plasmids from *E. coli*^26^. Because PtNPs used in the present study were spherical, as evidenced by TEM imaging (Supplementary Fig. 1), they are unlikely to intercalate with plasmid DNA and inhibit its replication. Hence we explored whether they would inhibit *E.coli* gyrase using commercial gyrase drug testing kit (Topogen, USA). Our results (Fig. 6 and Supplementary Fig. 15) revealed that relative to ciprofloxacin (interfacial gyrase poison) treatment, exposure to PtNP both with gyrase (Lane 5) and without gyrase (Lane 7), caused appearance of an extra smaller band at bottom of the gel, indicating PtNPs is likely to induce DNA cleavage either in presence or absence of gyrase, which along with compromised inner membrane integrity might account for plasmid curing ability of PtNPs.

## Discussion

Metal nanoparticles were widely reported to exhibit antibacterial effect^27–29^ and anti-biofilm effect^30–33^ few studies have also explored efflux inhibitory activity of metal NPs^34–36^. Reports on the ability of metal NPs to cure plasmids from bacterial cells are very scarce. Only one recent report has shown that silver nanoparticles (AgNPs) cures plasmid from different bacteria^37^ although the mechanism of plasmid curing was not explored and neither the plasmid curing ability was evaluated *in vivo*. Typically metal NPs are known to damage cell wall^38,39^, perturb membrane potential^40^, induce ROS^41,42^ and might cause spatio-temporal aggregation on *E. coli* cell wall and induce size dependent toxicity^43^. Earlier report from our group showed that PtNPs was non-toxic to zebrafish at 0.5 mM, which is 25 fold higher than the concentration employed in the present study and rescues zebrafish from infection^19^. Further studies revealed that PtNPs exhibits an immune modulatory role in zebrafish which prevents subsequent infection by the same bacteria^17^. Due to its non-toxic nature and unusual immune modulatory property exhibited by PtNPs, we set out to explore its ability to cure plasmid *in vitro* and *in vivo*.

Preliminary evaluations revealed that pectin capped PtNPs possessed weak antibacterial activity. A high MIC in turn implies a low therapeutic index and hence reduced potential to be employed as an antibacterial agent. Nevertheless their biological activity at sub MIC concentrations remains to be explored. Interestingly among compounds evaluated only PtNPs displayed the ability to cure plasmid from MDR *E. coli* strain, whereas conventional curing agents like SDS/acridine orange were ineffective, although they were able to cure plasmid from an uncapsulated *E. coli* strain (Supplementary Fig. 8). Capsular staining revealed that carbapenem resistant *E. coli* has a capsule (Supplementary Fig. 6), since most capsular polysaccharides are negatively charged, electrostatic repulsion might have prevented SDS from interacting efficiently with the bacterium. Thus relative to conventional plasmid curing agents, PtNPs’ plasmid curing ability was retained irrespective of capsular status of *E. coli*.

Earlier report has shown that nano alumina could enhance conjugative transfer of RP4 plasmid within bacteria and even across genera (between *E. coli* and *Salmonella spp*) by 200 fold, which was attributed to its ability to enhance expression of genes involved in mating pair formation, DNA replication and transfer^44^. Another recent report has also revealed that ZnO NP promoted competence, whereas TiO_2_ decreased competence, in *B. subtilis* during biofilm mode of growth^45^. Several other studies have also shown that nanoparticles can augment transformation efficiency in bacteria^46–48^. But studies on the ability of NPs to cure plasmid in bacteria are scarce^37^. To the best of our ‘knowledge, ours is the first report to show that PtNP mediated plasmid curing is possible *in vivo* in zebrafish. In addition, we have shown that at sub MIC levels, PtNP acts as an adjuvant along with meropenem to reduce bacterial bioburden in infected zebrafish by 2.4 log CFU relative to meropenem treatment alone (Fig. 4). The impact of PtNP on bacterial conjugation remains to be explored in future studies.

Plasmid cured strain exhibited slower growth and appeared as a small colony variant on agar plate, hence we reasoned that plasmid might possess some nutrient acquisition genes which prevented normal growth. Our growth fitness assay revealed that SCV could not compete with wild type by the end of day 7 (Supplementary Fig. 9). Previous report has shown that co-existence of mcr-1 and *bla*_NDM 5_ in plasmid leads to lower fitness and virulence in a clinical isolate of *E. coli*^49^. PCR amplification (Supplementary Fig. 4) showed that plasmid harbored *bla*_NDM_ but attempts to amplify mcr-1 by PCR using isolated plasmid DNA as a template was unsuccessful (Data not shown). Interestingly, plasmid cured strain displayed poor biofilm formation relative to wild type strain, although this appeared surprising, an earlier study has shown that IncI1 plasmid from enteroaggregative *E.coli* encodes a type IV pilus that contributes to plasmid conjugation, epithelial cell adherence and adherence to abiotic surfaces and pilS inactivation reduced biofilm formation on a glass slide by approximately 50%^50^. Identification of plasmid incompatibility types of U3790 plasmid using PCR based method revealed that plasmid encodes both type IV pili (IncI1) and Type F pili (IncFI and Inc FII) (Supplementary Table 2 and Supplementary Fig. 5) that enables initial attachment onto abiotic surfaces and hence plasmid loss leads to ~50% reduction in biofilm formation on glass surface.

A variety of curing agents have been reported in literature to cure plasmids, which include, ethidium bromide, acridine orange and quinacrine, all of which functions as a DNA intercalating agent and interferes with plasmid replication^51^. Other agents like SDS acts preferentially and it was proposed that SDS acts at pili wherein plasmids are predominantly located on F+ cells^12^. Novobiocin and Coumermycin were also shown to cure drug resistant plasmids from *S. aureus* by interfering with the function of DNA gyrase which is required for proper replication and maintenance of plasmid. RNA polymerase inhibitor rifampicin was also shown to cause plasmid loss in rifampicin sensitive cells but not resistant cells^51^. Among the plant metabolites, plumbagin was shown to cure plasmid from drug resistant *E. coli* although mechanism of curing was not explored in that study^14^. In another study, 8-epidiosbulbin E acetate (EEA) (norditerpene) was shown to cure plasmid from diverse bacteria including vancomycin resistant enterococci thereby reversing drug resistance in MDR bacteria^52^. Of late, plasmid incompatibility is widely exploited to cure plasmids in diverse bacteria^53,54^. In one such recent study, conjugative interference plasmids devoid of toxins and antibiotic resistance gene was shown to cure antibiotic resistant plasmids from different strains of *Enterobacteriaceae in vitro* and restored antibiotic susceptibility in mouse gut population where this resistance trait has spread^55^. It was also shown that mutation of partitioning genes alone was sufficient to cause plasmid loss in bacteria^56^.

An earlier report has shown that Cis dichlorodiamine platimum (II) chloride but not platinum salt K_2_PtCl_4_ was effective in curing *E. coli* of high copy number plasmids and the authors had proposed square planar nature of the compound facilitates its interaction with supercoiled/nicked plasmid DNA as an intercalating agent, thereby preventing plasmid replication and concomitantly for the loss of plasmid during segregation to daughter cells^57,58^ The same group also reported mixed ligand complexes of Platinum (II) were effective in curing Col E1 plasmids from *E. coli*^26^. In the present study, PtNPs were spherical (Supplementary Fig. 1 and Fig. 5) and not planar, and hence intercalation with plasmid DNA may not be cause for plasmid loss during segregation. In addition PtNP at the concentration employed for curing did not induce ROS (Supplementary Fig. 13) and neither affected outer membrane permeability nor the membrane potential (Supplementary Fig. 12). Our TEM imaging revealed that PtNPs could penetrate through capsule and interacted with cell surface without causing damage to the cell wall (Fig. 5) and at sub MIC levels, PtNPs were shown to affect only membrane integrity (Table 5), which causes leakage of nucleotides without compromising the viability (Supplementary Fig. 14). Whereas, the positive control Triton-X-100 compromised inner membrane integrity, affected viability (Supplementary Fig. 14) and yet did not result in plasmid curing (Table 3). Another report has shown single square electric pulse of 10 J can cause loss of plasmid from bacterial cells, most likely by inducing transient holes in cell membranes that aid in plasmid loss^59^, which offers support to our observation that affecting inner membrane integrity might result in plasmid curing. Gyrase inhibition assay showed PtNPs induce plasmid DNA cleavage both in presence and absence of gyrase (Fig. 6 and Supplementary Fig. 15). Thus mechanistic studies indicated that a compromised inner membrane integrity coupled with cleavage of plasmid DNA, might account for plasmid curing effect of PtNPs. Earlier studies have shown that TiO_2_ NPs induce genotoxicity in rats by causing double stranded DNA breaks (gamma H2A-X-foci) and DNA deletions, both directly and indirectly, by triggering oxidative stress^60^. Similarly gold NPs (regardless of size) and Cerium dioxide (7 nm) were shown to cause DNA damage, which was primarily mediated by oxidative stress^61,62^. Another report has shown that ZnO NPs cause an increase in oxidative stress in a dose dependent manner, which activates the apoptotic pathway mediated by mitochondria and caspases^63^. We have shown that at the concentration tested PtNPs do not generate ROS (Supplementary Fig. 13) and hence DNA cleavage induced by PtNPs (Fig. 6) might be independent of oxidative stress. A recent review has comprehensively summarized importance of plasmid curing agents in restraining spread of antimicrobial resistant genes and has underscored need for non-toxic plasmid curing agent *in vivo*^64^. Our study shows that plasmid curing occurs *in vivo* at sub MIC levels of PtNPs, which is nontoxic as evidenced by hemolysis assay (Supplementary Fig. 10) and by lack of change in liver and brain enzyme profiles in zebrafish at 25X higher concentration than used in present study as reported earlier^19^. This opens up the possibility of exploring biogenic NPs as plasmid curing agents *in vivo* in higher animal models. Moreover, PtNPs in conjunction with meropenem significantly reduces bacterial bioburden in infected zebrafish (Fig. 4). By virtue of exerting anti-biofilm effect (Fig. 3), being non-toxic (Supplementary Fig. 10), affecting curing *in vivo*, PtNP has the potential to be used as an adjuvant to address plasmid mediated drug resistance *in vivo* in higher animal models.

## Materials and Methods

### Synthesis of Biogenic Platinum NPs

Pectin capped platinum NPs were synthesized essentially as reported earlier^19^. The synthesized NPs was tested for its stability and was characterized by TEM, Zetapotential and Particle size analyzer and it was found to corroborate with characteristics that was reported earlier^19^.

### Bacterial strains

*Escherichia coli* U3790 strain was obtained from tertiary care hospital in Chennai. Mg1655 strain of *E. coli* was used as the reference strain. Antimicrobial profile of *E. coli* strains against various antimicrobial agents were determined by microbroth two fold dilution method (CLSI).

Plasmid extraction was performed using Favor prep plasmid extraction mini kit (Favorgen, Biotech Corp, Taiwan) as per manufacturer’s instructions. Extracted plasmid DNA was run on 1.5% agarose gel along with standard molecular weight markers.

### Evaluation antibacterial and plasmid curing potential of platinum NPs

MIC of PtNPs were evaluated by standard two fold microbroth dilution method^65^. The effect of various concentration of PtNPs in curing plasmid from U3790 strain was checked by plating NPs treated mixture onto LB agar plates followed by replica plating/ spot plating of grown colonies on LB agar containing meropenem (4 µg/ml). Colonies that show up on both LBA and LBA + antibiotic retain their plasmids. Whereas those that fail to grow on antibiotic containing plates have lost their plasmids.

Cured strains were reevaluated for their MIC for ceftriaxone and meropenem by standard microbroth dilution method. Modified Hodge test was performed as per standard protocol^66^ for wild type, uncured and cured strain to prove loss of resistance to carbapenems in plasmid cured strain. All experiments were performed in triplicates as three independent experiments and represented as average with standard deviation from mean.

### PCR amplification of ESBL and mcr-1 genes

Primers sequences for *bla*_OXA-23_, *bla*_OXA-48_, *bla*_VIM_, *bla*_KPC_, *bla*_NDM-1_, mcr-1 were designed using primer3 software. The conditions for amplifications of these respective genes from the corresponding plasmids were as described earlier^67^.

### Biofilm formation

Ability of wild type and plasmid cured isogenic strain to form biofilms on glass surface was evaluated both by crystal violet assay^68^ and by qualitatively evaluating biofilms formed on glass slide by fluorescence microscopy.

### Zebrafish infection

The CPCSEA guidelines for laboratory animal facilities (Central Act 26 of 1982) were adhered in all *in vivo* experiments. The protocols were approved by Institutional Animal Ethics Committee (CPCSEA-493/SASTRA/IAEC/RPP) of SASTRA deemed University, India and experiments were performed by following protocols approved by Institutional Animal Ethics Committee, SASTRA deemed University, India. Toxicity analyses of PtNPs on zebrafish were carried out essentially as reported earlier^24^. Fish were injected intramuscularly with U3790 strain and 2 h post infection, fish were administered with sub MIC levels of PtNPs alone and NPs in combination with meropenem. Independently, meropenem treatment was also maintained. The concentration of meropenem was chosen such that only cured strains will get eliminated but not the plasmid harboring strain. After 48 h of treatment, fish were anaesthetized by ms-222, euthanized by decapitation and muscle tissue was dissected, homogenized and plated onto LB agar to determine plate counts.

### TEM imaging

In order to discern interaction of NPs with bacteria that possessed a capsule, sample preparation for TEM was performed without dehydration and fixing so that ability of PtNPs to penetrate capsule could be discerned. TEM imaging was performed using Transmission Electron Microscope (JEOL-JEM 1011, Japan).

### Membrane permeability

Change in membrane permeability due to NP treatment was evaluated using NPN. Outer membrane of gram negative bacteria displays reduced permeability to NPN. Substances that alter outer membrane permeability leads to increased partitioning of NPN to the outer membrane resulting in enhanced fluorescence which can be quantified as NPN uptake factor^69^.

### Membrane potential

Effect of sub MIC levels of PtNPs on membrane potential was discerned using fluorophore DiSc3 as reported earlier^70^. If membrane potential is intact, DiSc3 partitions to lipid bi layer of bacterial cells and fluorescence gets quenched. When the potential is disturbed, DiSc3 partitions to aqueous milieu resulting in enhanced fluorescence.

### Membrane integrity

Ability of sub MIC levels of PtNPs to compromise the inner membrane integrity was assessed by measuring leakage of nucleic acid and protein from treated cells by quantifying absorbance at 260 and 280 nm respectively. Triton X 100 was used as the positive control^71^. The experiment was performed as three independent experiments and represented as average with standard deviation from mean.

### ROS

We evaluated ability of sub MIC levels of PtNPs to induce ROS which in turn reduces fluorophore DCHF-DA to DHF was measured using spectrofluorimeter (Jasco FP-8200) as reported earlier^72^.

### Inhibition of DNA gyrase

Ability of PtNPs to inhibit DNA gyrase was evaluated using *E. coli* DNA gyrase drug screening kit (Cat No: TG2001G-1 kit) from TopoGen by following manufacturer’s instructions. Relaxed pHOT-1 plasmid DNA, Linearized pHOT-1 DNA, Supercoiled pHOT-1 DNA and Relaxed pHOT1 DNA with gyrase and Relaxed pHOT1 DNA with gyrase and Ciprofloxacin (Interfacial poison of gyrase) were used as controls. PtNPs with and without gyrase plus substrate (relaxed pHOT-1 DNA) were used as treatments. After incubation of reaction mixtures at 37 °C for 1 h, the reaction was stopped by extraction with 10% SDS followed by treatment with protease and reaction was terminated with chloroform isoamyl alcohol (24:1 ratio). The aqueous layer containing DNA from various treatments was loaded onto two 1% agarose gel and was electrophoresed at 50 V for1h followed by destaining of EtBr and was imaged using gel documentation system (BioRad, USA) and results were analyzed.

### Complementation studies

Plasmid was extracted from the wild type strain and was transformed into cured small colony variants by standard CaCl_2_ mediated transformation and the transformed colonies were selected on plates containing meropenem (4 µg/ml) wherein cured strains fail to grow. The transformants were further confirmed by increase in MIC for ceftriaxone and meropenem.

## Conclusion

In conclusion we have shown for the first time, biogenic PtNPs causes selective loss of plasmid from capsulated strain of *E.coli* U3790 strain which leads to a significant decline in MIC for meropenem and ceftriaxone. Plasmid loss results in formation of SCV that exhibits slower growth. Complementation of plasmid from wild type strain into cured strain restored drug resistant phenotype. Plasmid harbored *bla*_NDM_, *bla*_OXA-23_, *bla*_OXA-48_ genes which conferred resistant phenotype. Importantly, we have shown that NPs are non-toxic and are able to cause loss of plasmid *in vivo* and as an adjuvant along with meropenem, it caused a significant reduction in bacterial bioburden relative to meropenem treatment alone. Although it is clear that PtNPs do not function as an intercalating agent, it might cause plasmid loss by causing DNA cleavage and by perturbing inner membrane integrity. Further studies can shed light if it also affects conjugal transfer.

## Supplementary information

Supplementary information

## Data Availability

Almost all data generated or analyzed during this study are included in this published article (and its Supplementary Information files). Upon request raw data would be made available.
