# Supplementary material for: Sub lethal levels of platinum nanoparticle cures plasmid and in combination with carbapenem, curtails carbapenem resistant Escherichia coli
Source: Sci Rep. 2019 Mar 28;9:5305. doi: 10.1038/s41598-019-41489-3 (PMC6438982; doi:10.1038/s41598-019-41489-3)
Supplement: Supplementary file 1 — Supplementary information [file 41598_2019_41489_MOESM1_ESM.pdf]

Supporting Information:

Sub lethal levels of platinum nanoparticle cures plasmid and in combination with carbapenem, curtails carbapenem resistant *Escherichia coli*

Subhashree Bharathan[$,a], Niranjana Sri Sundaramoorthy[$a], Harini Chandrasekharan [a] Gagana Preetham[a], ArunKumar GaneshPrasad[a] Siva Bala Subramaniyan[b], Anbazhagan Veerappan[b] * and Saisubramanian Nagarajan*[a,b]

[$]Both authors have equally contributed for the work

[a] Center for Research in Infectious Diseases, School of Chemical and Biotechnology, SASTRA

[b] Department of Chemistry, School of Chemical and Biotechnology, SASTRA, Thanjavur

Supplementary Table 1: Antimicrobial profiling of XDR U3790

Antibiotic/Compound	Minimum Inhibitory Concentration (µg/ml)
**Ceftriaxone**	>128
**Ciprofloxacin**	256
**Colistin**	32
**Erythromycin**	256
**Gentamicin**	256
**Kanamycin**	>256
**Levofloxacin**	32
**Meropenem**	>32
**Norfloxacin**	>128
**Streptomycin**	16
**Tetracycline**	128
**Tigecycline**	>128
**Tobramycin**	128

Supplementary Table 2: Output from the plasmid finder tool used for identification of plasmid from the contig Sequences

Plasmid	Identity	Query / Template length	Contig	Position in contig	Note	Accession number
IncFIA	99.74	388 / 388	MyFirstAssembly_rep_c200	3592..3979		AP001918
IncFIB(AP001918)	98.39	682 / 682	MyFirstAssembly_rep_c201	5248..5929		AP001918
IncFII(pAMA1167-NDM-5)	99.62	261 / 261	MyFirstAssembly_rep_c213	7730..7990	pAMA1167-NDM-5	CP024805.1
IncI1	100	142 / 142	MyFirstAssembly_rep_c206	17954..18095	Alpha	AP005147
p0111	98.53	885 / 885	MyFirstAssembly_rep_c199	30512..31396		AP010962

Supplementary Figure 1: **TEM image and zeta potential of pectin capped PtNPs**

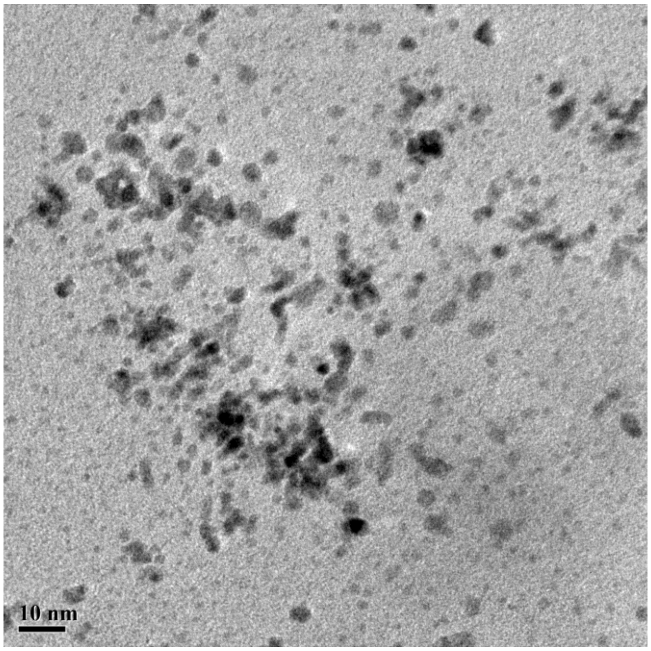

Results

			Mean (mV)	Area (%)	St Dev (mV)
**Zeta Potential (mV):**	-38.9	**Peak 1:**	-38.7	98.7	6.42
**Zeta Deviation (mV):**	6.74	**Peak 2:**	-59.8	1.3	2.50
**Conductivity (mS/cm):**	0.241	**Peak 3:**	0.00	0.0	0.00
**Result quality :**	Good				

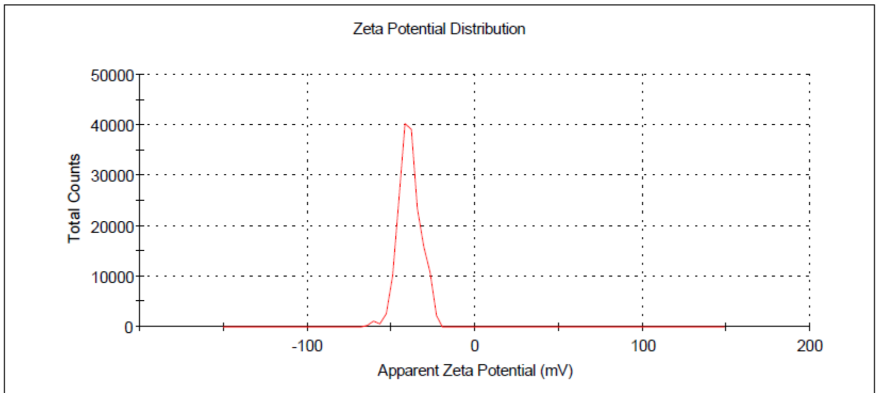

Supplementary Figure 2: (The full length gel image of Figure 1B) Gel picture showing absence of plasmid in cured SCV strain (L1, L5) and its presence in uncured strain (L2, L6) and in wild type (L3, L7), 1 KB ladder Molecular weight marker (L4).

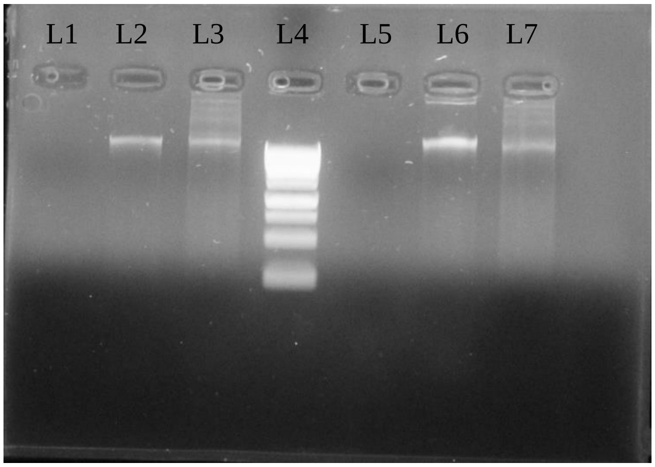

Supplementary Figure 3: **ABST test showing plasmid curing restores sensitivity to meropenem in SCV.** Antibiotic susceptibility test was performed for the wild type U3790, large colony variant (LCV) and small colony variant (SCV) by exposing the strain to PtNPs and the zone of inhibition was measured. SCV exhibited a larger zone of inhibition (16mm) relative to its wild type and LCV (10mm).

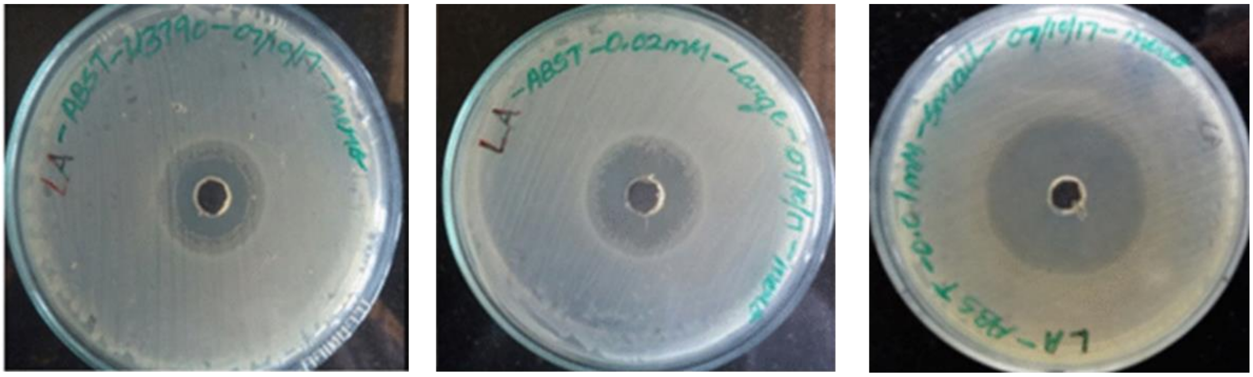

Wild type LCV SCV

Supplementary Figure 4: **PCR amplification of ESBL and mcr-1 genes.** Plasmid harbored carbapenem resistant genes**.** *bla*$_{OXA23}$, *bla*$_{OXA\ 48}$ and *bla*$_{NDM}$ genes were amplified by PCR using plasmid DNA as template.

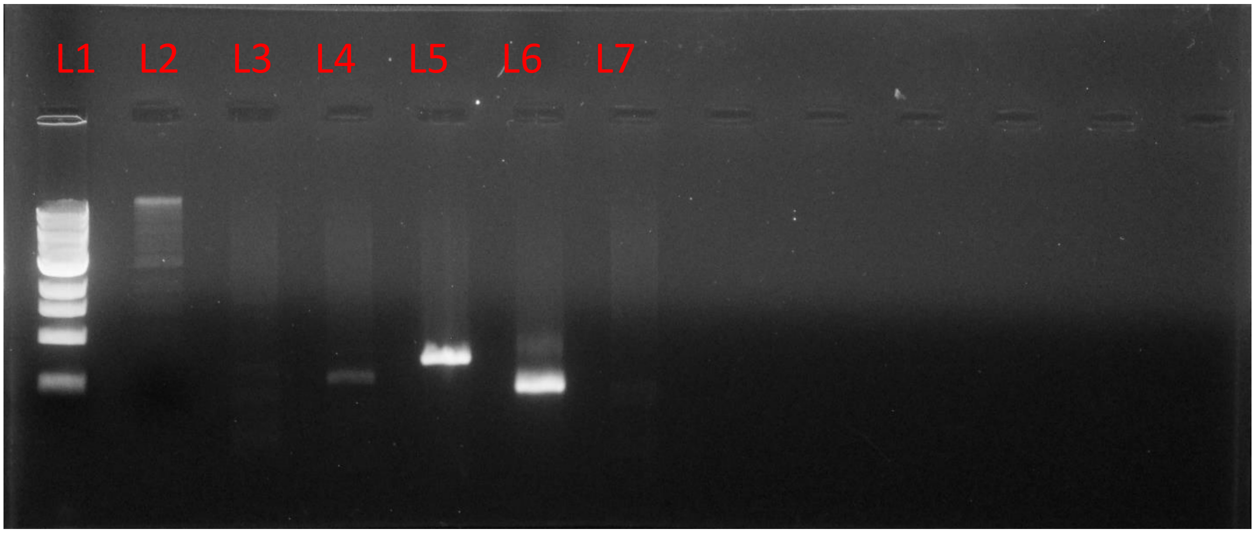

L1: Molecular weight marker
L2: Plasmid DNA template
L3: *bla*$_{KPC}$
L4: *bla*$_{OXA23}$
L5: *bla*$_{OXA48}$
L6: *bla*$_{NDM}$
L7: *bla*$_{VIM}$

Supplementary Figure 5: Detection of Incompatibility groups in plasmid. PCR amplification of plasmid from U3790 as template showed the presence of IncI1, IncFII, IncFIA, IncFIB and pO111.

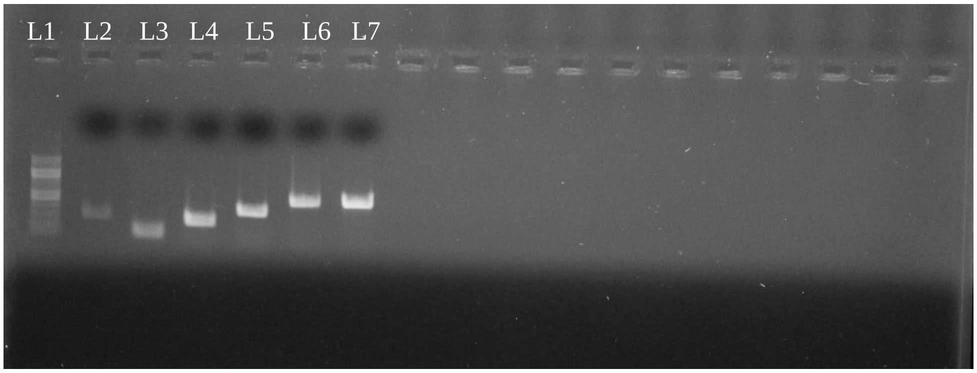

Lane Description:
L1: Molecular weight marker
L2: Other sample
L3: IncI1
L4: IncFII
L5: IncFIA
L6: IncFIB
L7: pO111

Supplementary Figure 6: **Capsulated strain**: Capsular staining indicates that U3790 possesses capsule as evidenced by halo around bacilli, which might have prevented the action of SDS/AO on plasmid.

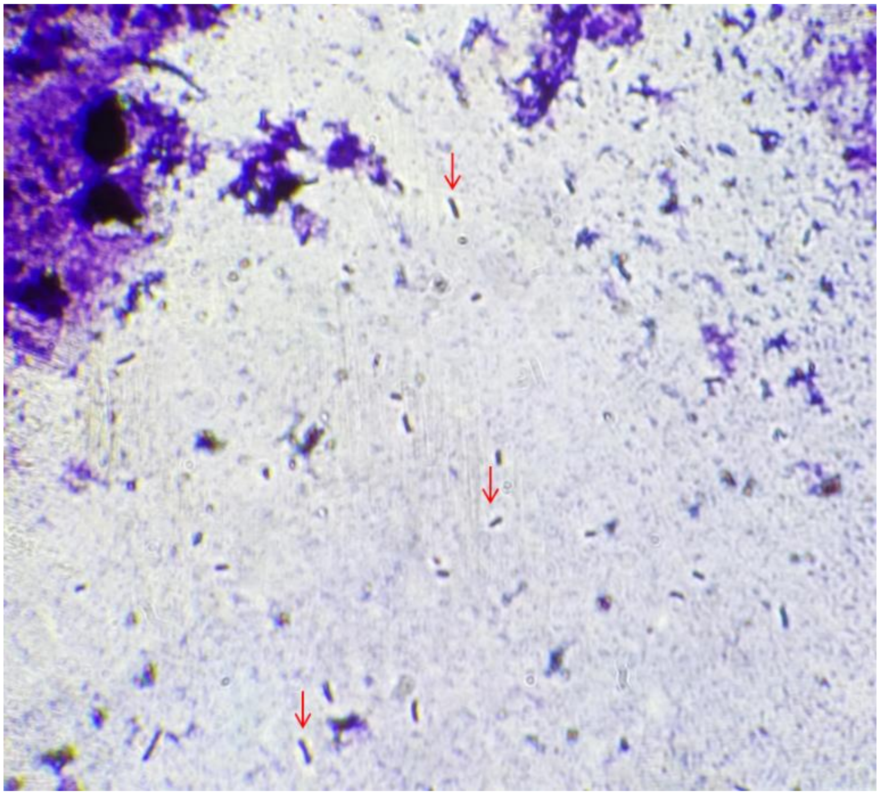

Supplementary Figure 7: (The full length gel image of Figure 2b) Plasmid extracted from transformants on LB + antibiotic plate showed reappearance of plasmid band.

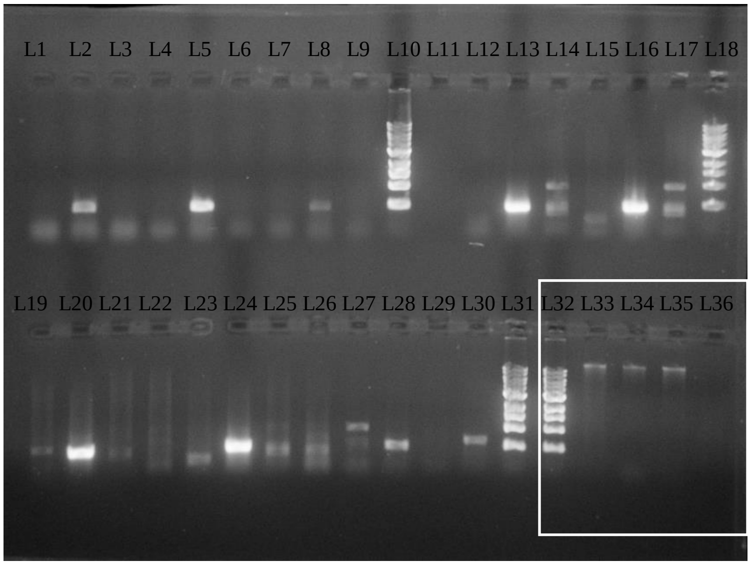

L1-L31 – Samples of different experiment

L32 - Molecular weight marker

L33 - U3790 Wild type

L34 - Transformant – 1

L35 – Transformant – 2

L36 – Untransformed cured colony (SCV)

Supplementary Figure 8: PtNPs cures plasmid from *E.coli* strain. 20 µM of PtNPs was effective in eliminating plasmid from *E.coli* DH5α strain harboring yeast expression vector p2GB42/Kan

Plasmid Gel Image

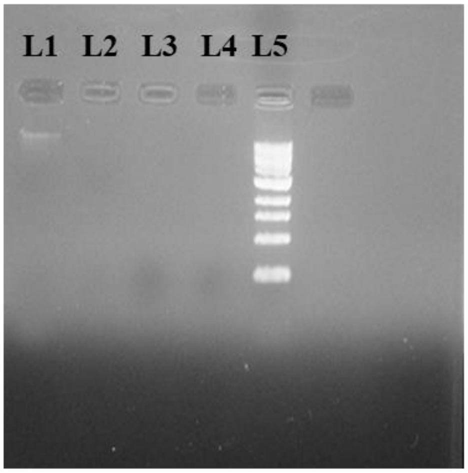

L1- Plasmid from *E.coli* 17260
L2- Plasmid from *E.coli* 17260 treated with Nanoparticle(0.02mM)
L3- Plasmid from *E.col*i 17260 treated with SDS
L4- Plasmid from *E.coli* 17260 treated with AO
L5- Molecular Weight Marker

Spot Plating on Kanamycin Plates

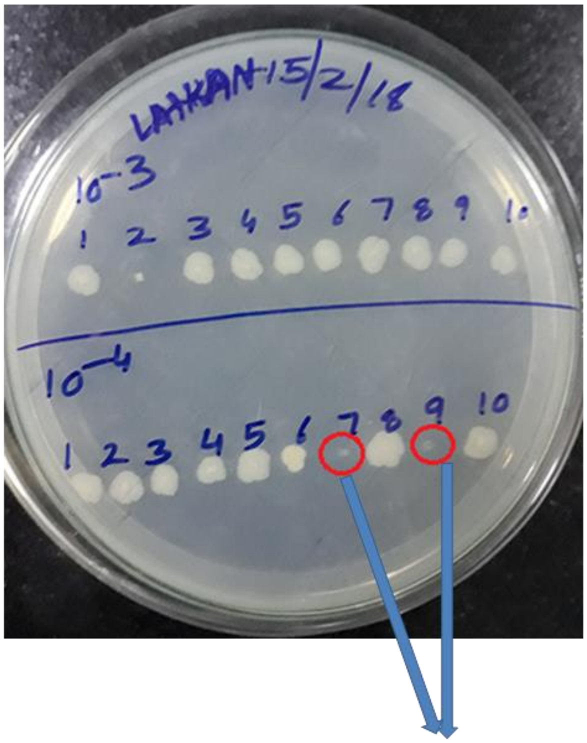

Cured Colonies

Supplementary Figure 9: Growth fitness assay revealed that SCV displayed lower fitness relative to LCV.

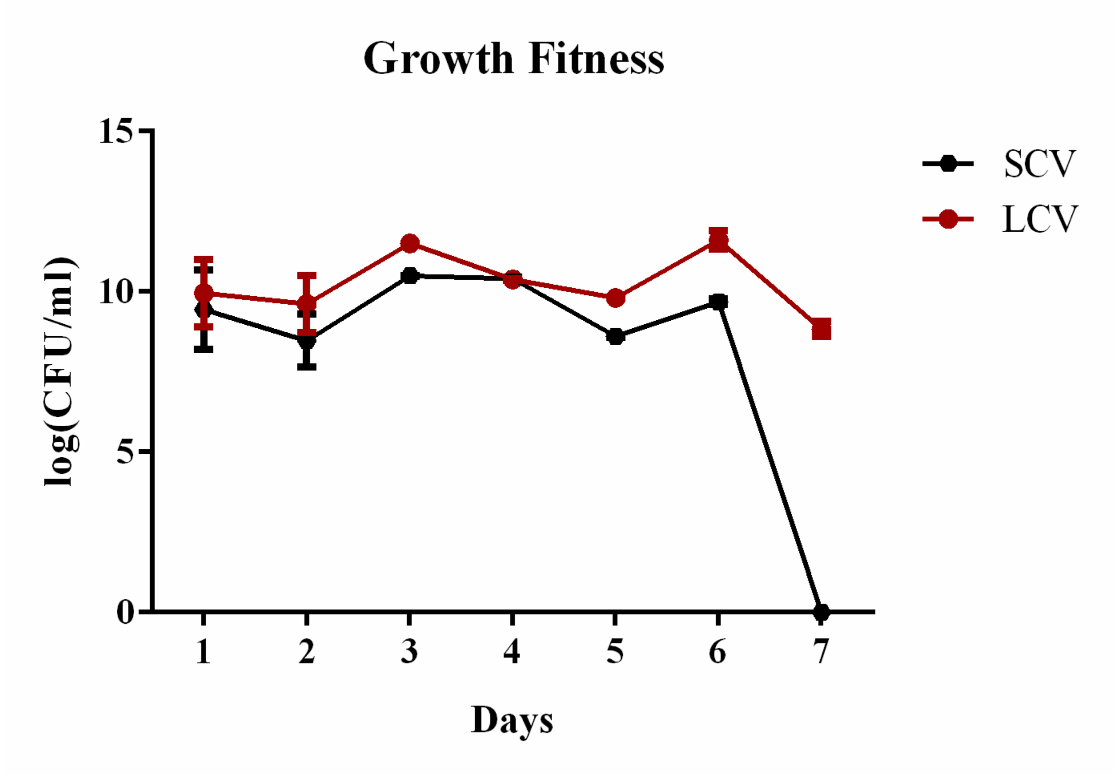

Supplementary Figure 10: Hemocompatibility assay. (A) Untreated human RBC and (B) human RBC treated with 200 µM PtNPs. RBC were incubated with 200µM PtNPs for 30min and evaluated under microscope for morphological changes. No damage to the cells were observed, which shows intrinsic hemocompatibility.

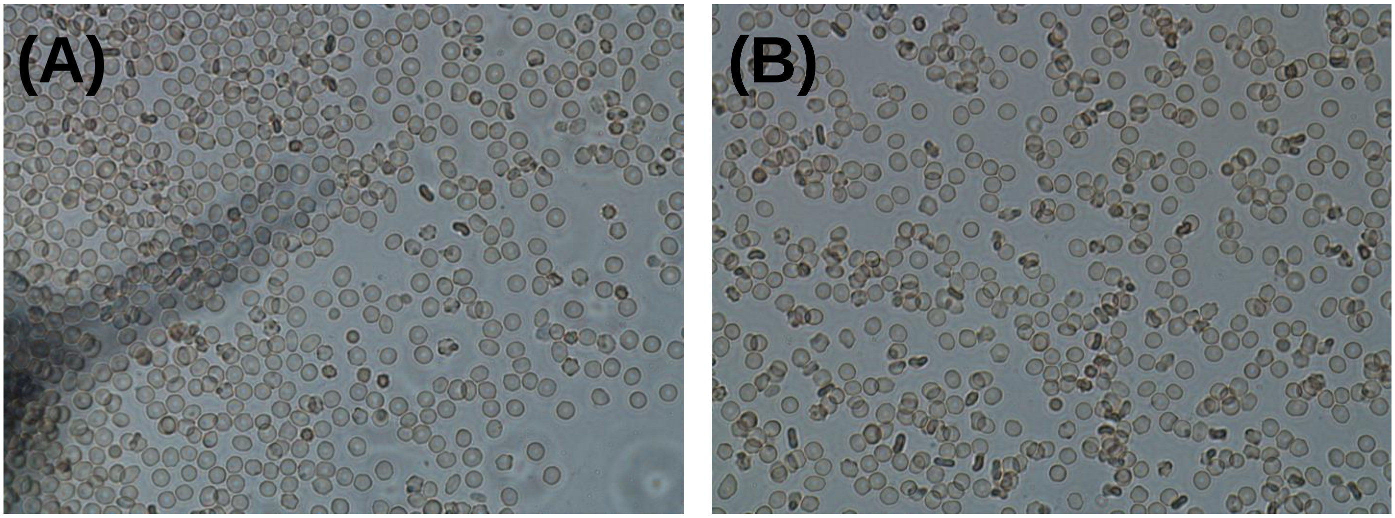

Supplementary Figure 11: **PtNP along with antibiotic cures plasmid *in vivo*.** SCVs isolated from infected muscle tissue of fish lacks plasmid Molecular weight marker (L6), SCV from zebrafish muscle tissue treated with PtNP alone (L7 & L8) and PtNP + Meropenem (L9&L10) were devoid of the plasmid relative to bacteria treated with meropenem (L11)

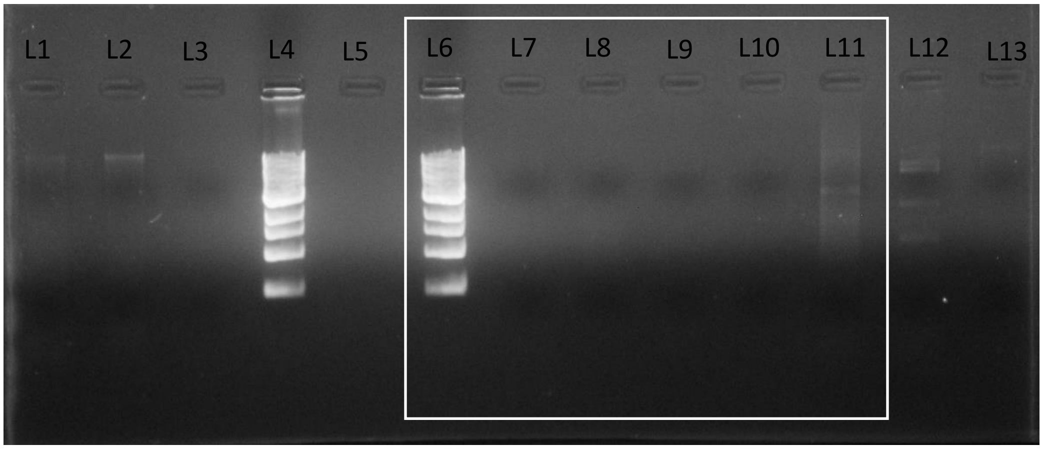

KEY:

L1 to L5 & L12, L13 - Samples of different experiment

L6 - Molecular weight Marker

L7 & 8 - SCV from zebrafish muscle treated with PtNP alone

L9 & 10 - SCV from zebrafish muscle treated with Meropenem +PtNPs

L11 – SCV from zebrafish muscle treated with meropenem

Supplementary Figure 12: **PtNPs does not perturb Membrane potential in carbapenem resistant *E.coli*.** Cells were incubated with PtNPs and Disc3 for 1hr and the fluorescence were measured at Ex605nm and Em665nm. The experiment was performed in triplicates and the error bar represents the mean standard error from three independent experiments.

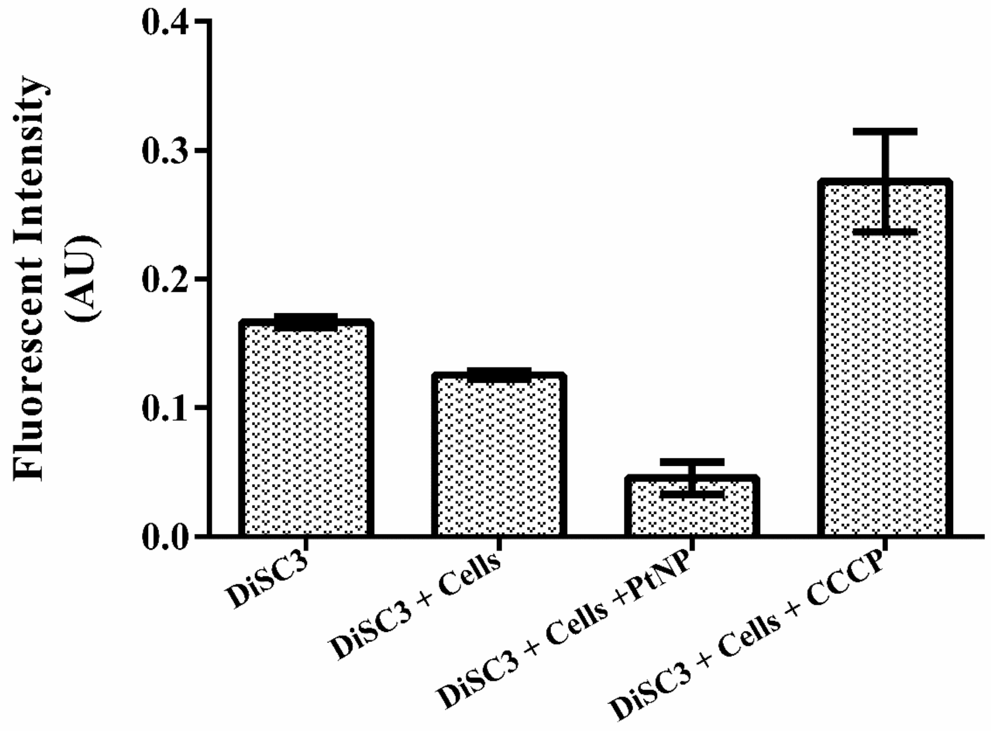

Supplementary Figure 13: **PtNPs at sub MIC levels does not induce intracellular ROS in *E.coli.*** Cells were treated with PtNPs and H_2O_2, as positive control. The fluorescence intensity is measured with excitation and emission at 495nm and 529nm. ROS generation was determined by the conversion of DCFDA to DCF. The experiment was performed in triplicates and the error bar represents the mean standard error from three independent experiments.

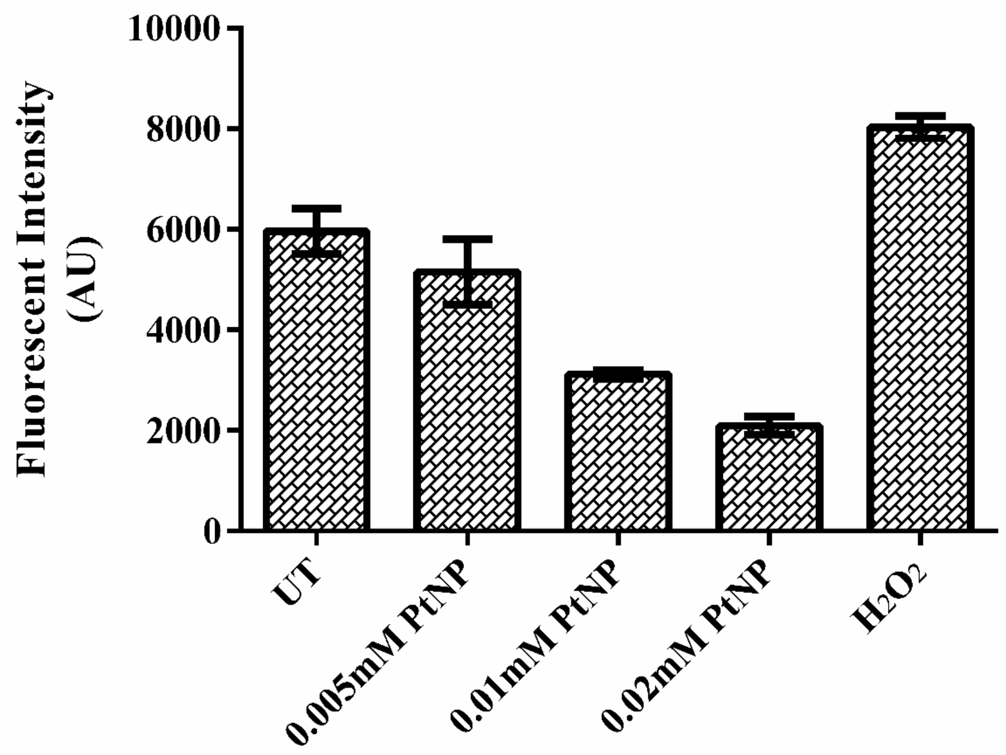

Supplementary Figure 14: Platinum NP (20 μM) treatment for 24h does not affect cell viability of *E.coli*. Proportion of propidium iodide stained cells were negligible in PtNP treatment relative to Triton X 100 treatment and plating reveals reduction in cell density for Triton X 100 treatment but not PtNP treatment.

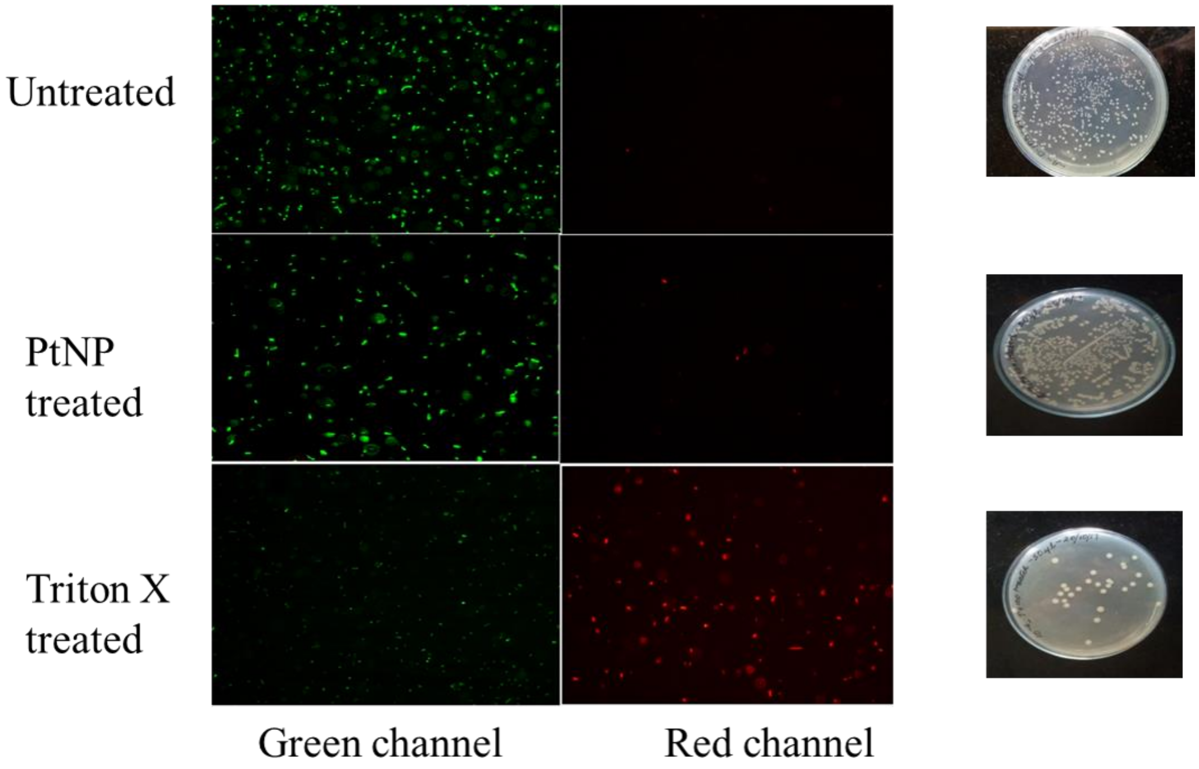

Supplementary Figure 15: (The full length gel image of Figure 6) PtNPs induce DNA cleavage in presence and absence of gyrase.

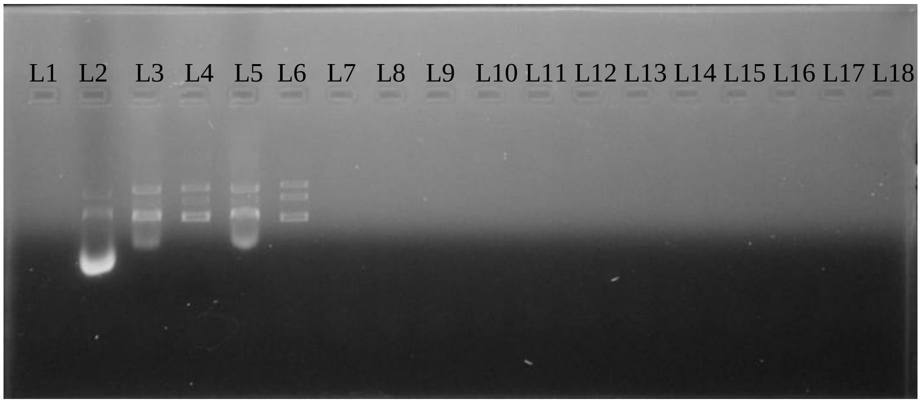

L2 – R. DNA + gyrase+ ciprofloxacin

L3 – R. DNA + gyrase+ PtNPs

L4 – R. DNA + gyrase

L5 – R. DNA + PtNPs

L6 – R. DNA

L1, L7 – L18 – Empty wells
